# Daptomycin-Induced Acute Eosinophilic Pneumonia: Late Onset and Quick Recovery

**DOI:** 10.1155/2017/8525789

**Published:** 2017-11-01

**Authors:** Mohamad Rachid, Khansa Ahmad, Meghan Saunders-Kurban, Aelia Fatima, Aditya Shah, Anas Nahhas

**Affiliations:** Internal Medicine, Advocate Christ Medical Center, Oak Lawn, IL, USA

## Abstract

**Background:**

Daptomycin is a cyclic lipopeptide antibiotic that provides great coverage for gram positive cocci. From the early years of daptomycin use, concerns were raised regarding the pulmonary side effects of daptomycin and potential development of acute eosinophilic pneumonia (AEP) secondary to daptomycin therapy.

**Discussion:**

AEP could be idiopathic or induced by drugs or toxins. It is a distinct entity from atopic diseases and autoimmune, parasitic, or fungal infections that can also cause pulmonary eosinophilia. Multiple medications are associated with acute eosinophilic pneumonia. Multiple cases of daptomycin-induced AEP have been reported in the literature. Diagnosis of AEP is based on clinical history, laboratory tests, and radiographic studies. Obtaining bronchoalveolar lavage or lung biopsy is needed to confirm the diagnosis. Timing of the drug use and clinical presentation is crucial in the diagnosis of drug-induced AEP. Discontinuation of the offending drug and systemic corticosteroids are the mainstay treatment with great outcomes and recovery.

**Conclusion:**

We present a case of AEP caused by daptomycin, with complete recovery after discontinuation of daptomycin and administration of steroids. The patient had AEP after almost 6 weeks of daptomycin therapy which has never been reported in literature and our patient achieved complete recovery with appropriate management.

## 1. Introduction

Daptomycin is a cyclic lipopeptide antibiotic approved by the FDA in 2003. Daptomycin provides great coverage for gram positive cocci. Shortly after its approval, concerns were raised regarding the pulmonary side effects of daptomycin and potential development of acute eosinophilic pneumonia (AEP) secondary to daptomycin therapy. About 35 cases were reported in the literature. We present a case of AEP caused by daptomycin, with complete recovery after discontinuation of daptomycin and administration of steroids. The patient had AEP after almost 6 weeks of daptomycin therapy which has not been reported in the literature. The patient achieved complete recovery in less than 48 hours of appropriate management.

## 2. Case Report

A 64-year-old male, with past medical history of peripheral arterial disease, had a left groin graft placement that was complicated with Methicillin Resistant* Staphylococcus Aureus* (MRSA) infection. Intravenous vancomycin was initiated which was complicated by the red man syndrome. Vancomycin was discontinued and the patient was switched to intravenous daptomycin 6 mg/kg/day for six weeks.

After almost six weeks of daptomycin therapy, the patient presented with progressive shortness of breath of two-day duration with associated fever and productive cough. Initial vital signs upon presentation were temperature 38.6°C (101.5°F), heart rate 100 beats per minute, respiratory rate 22 breaths per minute, oxygen saturation 93% on room air, and blood pressure 92/66 mmHg. Chest exam revealed coarse rhonchi bilaterally.

The initial white blood cells count was 18.0 K/UL, elevated eosinophil count 1 K/UL, and elevated erythrocyte sedimentation rate (ESR) 60 mm/hr. Initial chest-X-ray showed acute bilateral interstitial infiltrates.

**Figure 1:**
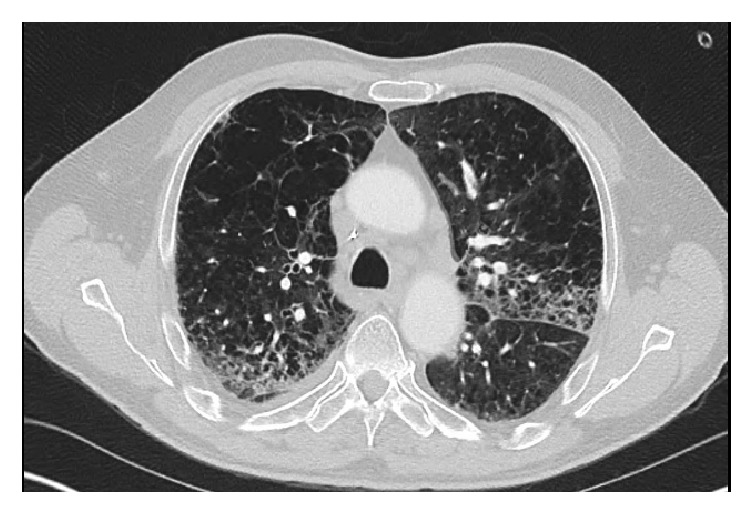
Chest Computed Tomography (CT) angiogram obtained to rule out pulmonary embolism (PE) showed diffuse bilateral pulmonary infiltrates, mediastinal lymphadenopathy, and small bilateral pleural effusions.

**Figure 2:**
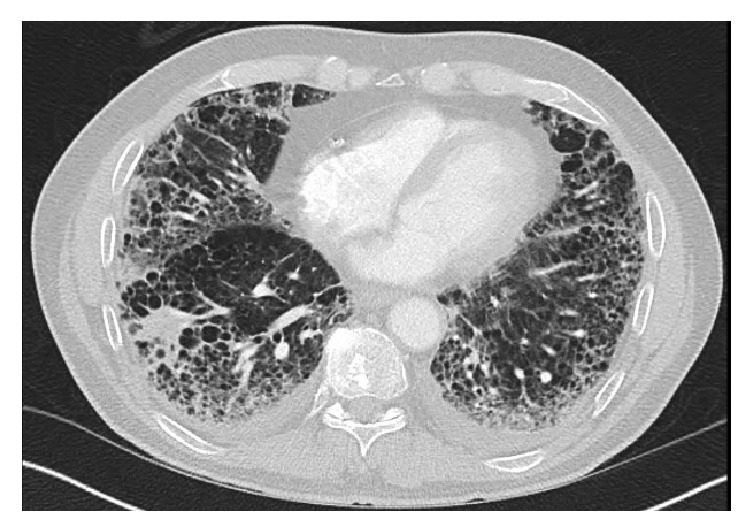
Chest Computed Tomography (CT) angiogram obtained to rule out pulmonary embolism (PE) showed diffuse bilateral pulmonary infiltrates, mediastinal lymphadenopathy, and small bilateral pleural effusions.

The patient was admitted for presumed hospital acquired pneumonia (HCAP) and started on intravenous piperacillin-tazobactam and levofloxacin. However, his clinical condition did not improve. He had progressive cough and increasing oxygen requirements. Chest Computed Tomography (CT) angiogram obtained to rule out pulmonary embolism (PE) showed diffuse bilateral pulmonary infiltrates, mediastinal lymphadenopathy, and small bilateral pleural effusions (Figures 1 and 2). The study was negative for PE. Suspicion for daptomycin-induced eosinophilic pneumonia was raised. Daptomycin was discontinued and intravenous methylprednisolone 80 mg every 8 hours was started.

**Figure 3:**
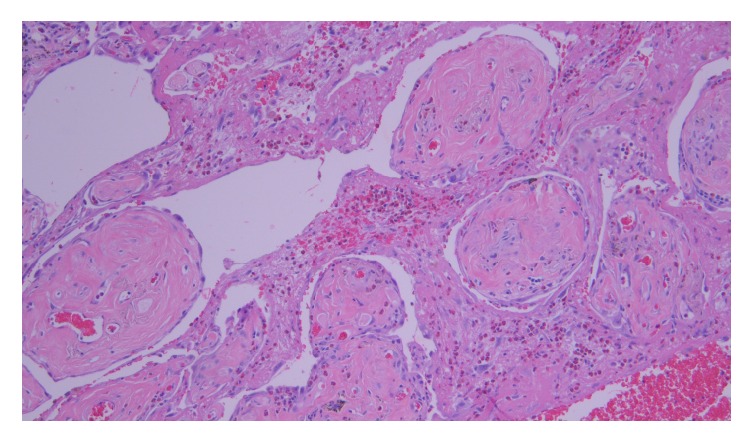
Picture of the lung tissue obtained showing chronic inflammatory changes, dense fibrinous airspace exudates, areas of organization, and prominent eosinophils, consistent with eosinophilic pneumonia.

Bronchoscopy to obtain bronchoalveolar lavage could not be done due to high oxygen requirements the patient needed and the patient was not intubated at that point to do it safely. Thus, the patient was sent for open lung biopsy via video assisted thoracoscopic surgery (VATS). The lung biopsy revealed chronic inflammatory changes, dense fibrinous airspace exudates, areas of organization, and prominent eosinophils, consistent with eosinophilic pneumonia (Figure 3).

**Figure 4:**
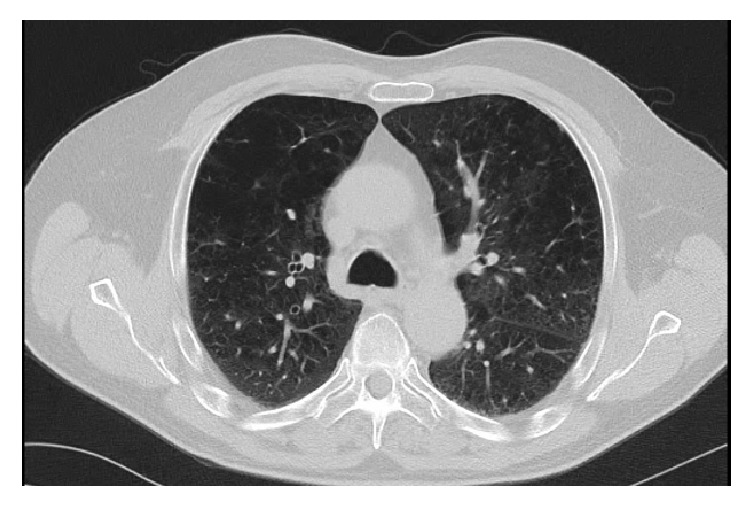
Repeat Chest CT, after one week of the initial CT, showing almost complete resolution of previous infiltrates, disappearance of mediastinal lymphadenopathy, and resolution of bilateral pleural effusions.

The patient improved within 48 hours after discontinuation of daptomycin and initiation of intravenous methylprednisolone. The patient was oxygenating well on room air on day 2 of steroids therapy. IV methylprednisolone was tapered gradually to oral prednisone 40 mg daily then prednisone was stopped in five days. Repeat Chest CT, after one week of the initial CT, showed almost complete resolution of previous infiltrates, disappearance of mediastinal lymphadenopathy, and resolution of bilateral pleural effusions (Figure 4). The left groin wound was well healed upon discharge.

## 3. Discussion

AEP is a subtype of acute pneumonia which is idiopathic or induced by drugs or toxins. It is distinct from atopic diseases and autoimmune, parasitic, or fungal infections that can also cause pulmonary eosinophilia. Multiple medications are associated with acute eosinophilic pneumonia including nonsteroidal anti-inflammatory drugs, minocycline [1], antidepressants, and antipsychotics [2].

Daptomycin is a cyclic lipopeptide antibiotic which was approved by the FDA in 2003. Daptomycin inhibits DNA, RNA, and protein synthesis by causing depolarization of the membrane potential when it binds the bacterial membrane [3]. It has excellent spectrum for gram positive bacteria [4]. Daptomycin is usually used against Methicillin Resistant* Staphylococcus Aureus* (MRSA) as a second line agent after vancomycin in treating skin, soft tissue infections, and bacteremia [5].

Early after the approval of daptomycin by the FDA, multiple cases of daptomycin-induced eosinophilic pneumonia have been reported. Hayes Jr. et al. reported the first case of daptomycin-induced eosinophilic pneumonia in 2007 [6]. Kim and colleagues reviewed the reported cases in the FDA Adverse Event Reporting System (AERS) submitted from 2004 to 2010. They put forth criteria to classify these cases as definite, probable, possible, or unlikely [7]. Based on their criteria, our patient had a definitive diagnosis of daptomycin-induced eosinophilic pneumonia.

Diagnosis of daptomycin-induced AEP is based on clinical history, laboratory results, and radiographic findings. Usually these patients present with dyspnea and cough with or without fever. The clinical presentation may vary from mild respiratory complaints to minimal requirement of oxygen, up to acute respiratory distress syndrome (ARDS). Laboratory workup may reveal peripheral eosinophilia, leukocytosis, or elevated markers of inflammation such as erythrocyte sedimentation rate (ESR) and C-reactive protein (CRP). Chest X-ray reveals bilateral pulmonary infiltrates. Chest CT may reveal bilateral consolidation, infiltrates, pulmonary nodules [3], ground glass opacity [8], and bilateral pleural effusions [2]. In our case, bilateral peripheral infiltrates were abundant which is consistent with eosinophilic pneumonia. Definitive diagnosis necessitates bronchoalveolar lavage (BAL) to obtain cell count. BAL with more than 25% of eosinophilic is diagnostic of eosinophilic pneumonia. Sometimes, a lung biopsy is needed to confirm the diagnosis. It is crucial to exclude other causes of eosinophilic lung disease such as parasitic infections, fungal infections, or vasculitis, before making the final diagnosis of any drug-induced AEP, including daptomycin. The Department of Pulmonary and Intensive Care at University Hospital in Dijon, France is maintaining a helpful website to report medications and drugs associated with lung injuries. The website is: http://www.pneumotox.com [9].

The criteria to diagnose AEP consist of 4 components: presence of febrile illness of less than 5-day duration, diffuse bilateral pulmonary infiltrates, hypoxemia defined as partial pressure of oxygen of less than 60 mmHg or oxygen saturation less than 90% on room air, and bronchoalveolar lavage with greater than 25% eosinophils or eosinophilic pneumonia on lung biopsy [10]. Solomon and Schwarz created criteria to diagnose drug-induced AEP, consisting of (1) presence of AEP confirmed by criteria mentioned earlier, (2) presence of causative drug with appropriate temporal relationship, (3) other causes of AEP excluded like fungal or parasitic infections, (4) clinical improvement after cessation of the drug, and (5) the recurrence of AEP with rechallenge to the drug [11]. Rechallenging with the drug is not recommended.

In Idiopathic AEP, most of the time, the peripheral eosinophil count is not elevated in the early phases but may become elevated later on during the course of the disease [12]. Our patient did have peripheral eosinophilia. We reviewed 28 reported cases of daptomycin-induced AEP and we found that 23 out of 28 cases did have peripheral eosinophilia. Peripheral eosinophilia is not necessary to diagnose AEP and it is not part of the current diagnostic criteria [10] but peripheral eosinophilia may suggest eosinophilic pneumonia. Yusuf et al. [13] suggested using the peripheral eosinophils count as a marker to monitor for daptomycin-induced AEP. They suggested obtaining baseline eosinophils count before starting daptomycin therapy then following up the count during treatment course to monitor for peripheral eosinophilia.

The pathophysiology of daptomycin-induced AEP is unclear. Daptomycin binds the lung surfactant irreversibly and may act like an antigen taken up by alveolar macrophages which then leads to inflammatory cascade and tissue damage. Alveolar macrophages present the antigen to T-helper 2 lymphocytes, which release multiple inflammatory factors like IL-5 and Eotaxin. IL-5 is known to induce production of eosinophils in bone marrow and recruit them to inflammatory sites. Eotaxin is a chemokine and acts as a chemoattractant which causes migration and recruitment of eosinophils [14, 15]. In case this theory is truly the cause of AEP in patients on daptomycin therapy, the question is why are only some patients affected by AEP on daptomycin therapy? Why is it uncommon to see this side effect from daptomycin therapy? These questions have yet to be answered with further research.

The histologic findings of drug-induced eosinophilic pneumonia have been described in multiple case reports. Most of the reported cases diagnosed daptomycin-induced AEP based on BAL findings. In cases where lung biopsies were obtained, the majority revealed organizing pneumonia with eosinophilic infiltrates and chronic inflammatory changes [3, 14, 16]. In our case, we confirmed the diagnosis by lung biopsy. Hayes Jr. et al. [6] reported alveolar and interstitial eosinophils with minimal edema in the biopsy results. Our patient's lung biopsy revealed chronic inflammatory changes, diffuse fibrinous airspace exudates, and prominent admixed eosinophils.

The mainstay treatment of daptomycin-induced AEP, and any other drug-induced AEP, is to give corticosteroids and to stop the offending drug. This practice is based on previous case reports of daptomycin-induced AEP and other medication induced lung injuries. Steroids are recommended to patients with acute lung injuries caused by amiodarone, cocaine, and bleomycin [17]. Our patient had a dramatic recovery within 36 hours after stopping daptomycin and starting steroid therapy. In most reported cases, daptomycin was stopped and patients received steroid therapy. In a few cases, daptomycin was stopped and steroids were not given. All of these patients had a complete recovery without relapse [3, 8, 13, 14, 18]. AEP is an acute inflammatory process and giving steroids is a reasonable treatment option. With reported cases of complete recovery by discontinuing daptomycin alone, is giving steroids warranted or not? There are no guidelines that address this question and further research is needed. However, it is reasonable to give steroids in patients with severe hypoxemia and respiratory failure. A systematic review was recently published on 35 reported cases in the literature [19].

## 4. Conclusion

Daptomycin is not widely used, yet physicians should be aware of daptomycin-induced AEP in any patient actively on daptomycin and presenting with respiratory complaints. Daptomycin-induced AEP is not related to therapy timing. It may occur as early as day three of treatment and as late as week six of the treatment course. AEP is a serious side effect of daptomycin but easily treatable if diagnosed early during the course of the disease. High clinical suspicion and thorough workup are needed for diagnosis of AEP. The current mainstay therapy is stopping daptomycin with or without steroid therapy. Further research is needed to assess the role of steroids in the treatment of AEP, including the optimal dose and duration of therapy.

This case is unique because the patient had daptomycin-induced AEP after almost six weeks of daptomycin therapy, which has not been previously reported in the literature. As long as the patient is undergoing daptomycin therapy, the risk of AEP still exists, even after a considerable amount of time has passed.
